# Effect of Preoperative Creatinine Levels on Mortality after Coronary Artery Bypass Grafting Surgery: an Observational Study

**DOI:** 10.21470/1678-9741-2018-0261

**Published:** 2019

**Authors:** Marcos Aurélio Barboza de Oliveira, Carlos Alberto dos Santos, Antônio Carlos Brandi, Ana Helena Dotta, Paulo Henrique Husseini Botelho, Moacir Fernandes de Godoy, Domingo M. Braile

**Affiliations:** 1 Hospital Amecor, Cuiabá, MT, Brazil.; 2 Hospital Femina Cuiabá, MT, Brazil.; 3 Hospital de Base São José do Rio Preto (HB), São José do Rio Preto, SP, Brazil.

**Keywords:** Kidney - Physiopathology, Coronary Artery Bypass, Risk Factors, Creatinine - Blood, Treatment Outcome

## Abstract

**Introduction:**

Renal function is an independent risk factor for mortality among on-pump coronary bypass grafting (ONCABG) patients. This association is well known in the international literature, but there is a lack of knowledge of how admission creatinine (AC) levels modulate each cardiovascular risk factor.

**Objective:**

The aim of this paper was to assess the effect of different AC levels on mortality among ONCABG patients.

**Methods:**

1,599 patients who underwent ONCABG between December 1999 and February 2006 at Hospital de Base in São José do Rio Preto/SP-Brazil were included. They were divided into quartiles according to their AC levels (QI: 0.2 ≤AC < 1.0 mg/dL; QII: 1.0 ≤ AC < 1.2 mg/dL; QIII: 1.2 ≤ AC < 1.4 mg/dL; and QIV: 1.4 ≤ AC ≤ 2.6 mg/dL). Seven risk factors were then evaluated in each stratum.

**Results:**

Mortality was higher in the QIV group than QI or QII groups. Factors such as age (≥ 65 years) and cardiopulmonary bypass (CPB) time (≥ 115 minutes) in QIV, as well preoperative hospital stay (≥ 5 days) in QIII, were associated with higher mortality rates. Creatinine variation greater than or equal to 0.4 mg/dL increased mortality rates in all groups. The use of intra-aortic balloon pump and dialysis increased mortality rates in all groups except for QII. Type I neurological dysfunction increased the mortality rate in the QII and III groups.

**Conclusion:**

Creatinine levels play an important role in ONCABG mortality. The combination of selected risk factors and higher AC values leads to a worse prognosis. On the other hand, lower AC values were associated with a protective effect, even among elderly patients and those with a high CPB time.

**Table:** 

Abbreviations, acronyms & symbols				
95% CI	= 95% confidence interval		EOL	= Extreme outliar
AC	= Admission creatinine		IAB	= Intra-aortic balloon
CABG	= Coronary artery bypass grafting		ICU	= Intensive care unit
CPB	= Cardiopulmonary bypass		IQI	= Interquartile interval
Cr Var	=Creatinine variation		MACCE	= Major adverse cardiac and cerebrovascular events
CV	= Cardiovascular		ONCABG	= On-pump coronary artery bypass grafting
CVD	= Cardiovascular disease		OR	= *Odds ratio*
DATASUS	= Brazilian Department of Public Healthcare Data		Qs	= Quartiles

## INTRODUCTION

Cardiovascular diseases (CVDs) are the main cause of death in Brazil. According to the Brazilian Department of Public Healthcare Data (DATASUS), in 2014, there were 340,284 CVD-related deaths in the country, which represented approximately 23% of all deaths in that year. Out of all cases of CVD, 60% involved ischemic diseases and myocardial infarction^[1]^. The Brazilian government spent BRL$13.5 billion on healthcare in that same year, and approximately 2.5% of that amount was directly applied to coronary artery bypass grafting (CABG) procedures alone or in combination with another procedure^[1]^.

In an attempt to decrease the mortality rate and hospital costs of cardiovascular (CV) patients, a group of authors studied data from 132 centers from eight different countries in Europe to identify risk factors in which they could predict a patient's odds of dying. One of these initiatives, which is now used around the world, is known as the EuroSCORE^[2]^. Although EuroSCORE has been able to accurately predict mortality rates for European patients, it has failed when applied to Brazilian patients^[3]^.

Lisboa et al.^[4]^ evaluated the applicability of EuroSCORE II at their institution and concluded that EuroSCORE II could not accurately foresee mortality rates for their patients; they decided to use a local scoring system. Santos et al.^[5]^ assessed 1,628 on-pump coronary artery bypass grafting (ONCABG) patients and found seven (pre-, intra- and postoperative) mortality risk factors and established cutoff values for each of them for their local population. The behavior of these risk factors is still unknown in cases in which the patient has a different admission creatinine (AC) value, which is another important risk factor.

The aim of this paper was to assess the effect of different AC levels on mortality among ONCABG patients.

## METHODS

This study was approved by the local Ethics Committee under number 454,518, and informed consent was waived due to the retrospective nature of the study.

From December 1999 to February 2006, 1,674 ONCABG patients from Hospital de Base of the city of São José do Rio Preto, São Paulo state, Brazil, were consecutively enrolled. To mitigate bias, patients with incomplete follow-up information who had undergone emergency surgery or who died during the surgery were excluded; a total of 46 patients (2.7%) were therefore excluded from the initial group.

We evaluated 1,628 patients and considered the following variables: use of intra-aortic balloon (IAB), cardiopulmonary bypass (CPB) time, creatinine variation, dialysis, type I neurological lesion, preoperative hospital stay, and age. The definitions and explanations of these factors are as follows:

- IAB: represents the whole number of implanted IABs, with no distinction regarding the time of placement (pre- or intraoperative);
- Creatinine variation: difference between AC and the highest creatinine value recorded in the intensive care unit (ICU);
- Type I neurological lesion: new and persistent focal motor deficit, coma, seizures or new encephalic lesion as determined by computed tomography or magnetic resonance imaging;
- Mortality: death by any cause within 30 days of surgery.

Extreme outliers were used to exclude patients with ACs greater than the established values, which were calculated using the following formula:

EOL=p75+3*IQR

where EOL=extreme outlier; p75=75^th^ percentile; and IQR=interquartile range.

The EOL value found was 2.6 mg/dL. Based on this value, 29 additional patients were excluded. The remaining 1,599 patients were separated into quartiles (Qs) based on their AC:

QI: 0.2 ≤ AC < 1 mg/dL;
QII: 1 ≤ AC < 1.2 mg/dL;
QIII: 1.2 ≤ AC < 1.4 mg/dL; and
QIV: 1.4 ≤ AC ≤ 2.6 mg/dL.

The risk factors and their respective cutoff points were extracted from the study by Santos et al.^[5]^ and were used to determine the mortality rate in each quartile. The risk factors and their cutoff points were: age (65 years), preoperative hospital stay (5 days), CPB time (115 minutes), creatinine variation (0.4 mg/dL), presence of IAB, dialysis and type I neurologic lesion.

### Statistical Analysis

Continuous and discrete variables were transformed into categorical data according to each cutoff value (0=below cutoff value; 1=above cutoff value and death=1; alive=0). Logistic regression analysis was performed for all of the risk factors, and the *P* value was obtained.

Other *P* values were calculated using Fisher's exact test. The type I error (α) was set at 5%, and all of the tests were two-tailed. The *odds ratio* (OR) and the 95% confidence interval (95% CI) were calculated. The statistical software used were GraphPad 3.0 and StatsDirect 1.9.15 for Windows^TM^.

The "n" value was not calculated due to the observational nature of the study.

## RESULTS

**Fig. 1:**
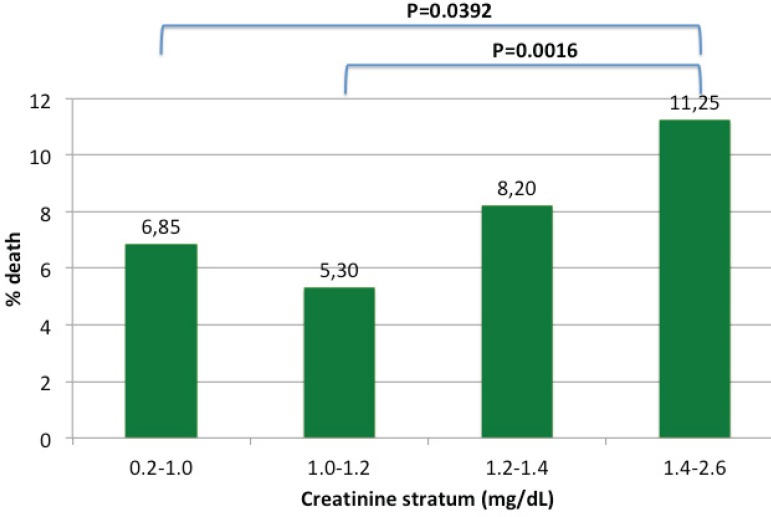
Inter-stratum mortality.

Mortality rates in each AC stratum are shown in Figure 1. The highest AC level-QIV-was associated with greater mortality when compared to AC values in the QI group or in the QII group (*P*=0.0392 and *P*=0.0016, respectively). Tables 1 to 4 show pre-, intra- and postoperative risk factors in each AC stratum.

**Table 1:** Mortality risk factors among ONCABG patients (n=336) with AC levels greater than or equal to 0.2 and less than 1 mg/dL (QI).

		Dead (23)	Alive (313)	*P*	OR (95% CI)
IAB	+	4	14	0.0169	5.00 (1.33-18.74)
	-	19	299		
CPB time	≥115 min	7	56	0.2775	1.79 (0.62-5.18)
	<115 min	16	257		
Cr var	≥0.4 mg/dL	13	61	0.0091	3.67 (1.38-9.78)
	<0.4 mg/dL	10	252		
Dialysis	+	4	1	0.0062	28.15 (2.58-307.22)
	-	19	312		
Neuro I	+	3	9	0.2077	2.87 (0.55-14.82)
	-	20	304		
Adm - surg	≥5 days	14	152	0.4442	1.48 (0.54-4.08)
	<5 days	9	161		
Age	≥65 years	12	112	0.3597	1.57 (0.59-4.14)
	<65 years	11	201		

AC=admission creatinine; Adm - surg=preoperative hospital stay; CI=confidence interval; CPB=cardiopulmonary bypass; Cr var=creatinine variation; IAB=intra-aortic balloon; min=minutes; Neuro I=type I neurological lesion; ONCABG=on-pump coronary artery bypass grafting; OR=*odds ratio*

**Table 2:** Mortality risk factors among ONCABG patients (n=396) with AC levels greater than or equal to 1 and less than 1.2 mg/dL (QII).

		Dead (21)	Alive (375)	*P*	OR (95% CI)
IAB	+	3	26	0.2297	2.73 (0.53-14.10)
	-	18	349		
CPB time	≥115 min	8	57	0.0997	2.78 (0.82-9.41)
	<115 min	13	318		
Cr var	≥0.4 mg/dL	12	58	0.0133	4.37 (1.35-14.06)
	<0.4 mg/dL	9	317		
Dialysis	+	1	1	0.068	17.36 (0.82-367.41)
	-	20	374		
Neuro I	+	10	4	<0.0001	73.55 (16.20-333.76)
	-	11	371		
Adm - surg	≥5 days	12	171	0.9538	1.03 (0.31-3.43)
	<5 days	9	204		
Age	≥65 years	10	118	0.7591	1.21 (0.35-4.14)
	<65 years	11	257		

AC=admission creatinine; Adm - surg=preoperative hospital stay; CI=confidence interval; CPB=cardiopulmonary bypass; Cr var=creatinine variation; IAB=intra-aortic balloon; min=minutes; Neuro I=type I neurological lesion; ONCABG=on-pump coronary artery bypass grafting; OR=*odds ratio*

**Table 3:** Mortality risk factors among ONCABG patients (n=378) with admission creatinine levels greater than or equal to 1.2 and less than1.4 mg/dL (QIII).

		Dead (31)	Alive (347)	*P*	OR (95% CI)
IAB	+	7	15	0.014	4.86 (1.37-17.21)
	-	24	332		
CPB time	≥115 min	12	65	0.094	2.19 (0.87-5.52)
	<115 min	19	282		
Cr var	≥0.4 mg/dL	16	57	0.0134	3.18 (1.27-7.99)
	<0.4 mg/dL	15	290		
Dialysis	+	5	1	0.0008	62.67 (5.55-707.74)
	-	26	346		
Neuro I	+	8	11	0.0038	5.85 (1.77-19.35)
	-	23	336		
Adm - surg	≥5 days	19	150	0.0182	3.05 (1.20-7.72)
	<5 days	12	197		
Age	≥65 years	21	121	0.0737	2.30 (0.92-5.77)
	<65 years	10	226		

AC=admission creatinine; Adm - surg=preoperative hospital stay; CI=confidence interval; CPB=cardiopulmonary bypass time; Cr var=creatinine variation; Neuro I=type I neurological lesion; IAB=intra-aortic balloon; min=minutes; ONCABG=on-pump coronary artery bypass grafting; OR=*odds ratio*

**Table 4:** Mortality risk factors among ONCABG patients (n=489) with admission creatinine levels greater than or equal to 1.4 and less than 2.6 mg/dL (QIV).

		Dead (55)	Alive (434)	*P*	OR (95% CI)
IAB	+	13	26	0.0013	3.88 (1.69-8.90)
	-	42	408		
CPB time	≥115 min	21	80	0.0039	2.60 (1.35-4.97)
	<115 min	34	354		
Cr var	≥0.4 mg/dL	35	151	0.039	2.00 (1.03-3.86)
	<0.4 mg/dL	20	283		
Dialysis	+	11	12	0.0062	4.05 (1.48-11.06)
	-	44	422		
Neuro I	+	5	18	0.6306	1.32 (0.41-4.20)
	-	50	416		
Adm - surg	≥5 days	32	228	0.5023	1.23 (0.66-2.30)
	<5 days	23	206		
Age	≥65 years	32	155	0.0101	2.27 (1.21-4.24)
	<65 years	23	279		

AC=admission creatinine; Adm - surg=preoperative hospital time; CI=confidence interval;CPB=cardiopulmonary bypass; Cr var=creatinine variation; IAB=intra-aortic balloon; min=minutes; Neuro I=type I neurological lesion; ONCABG=on-pump coronary artery bypass grafting; OR=*odds ratio*

When the risk factors of the QI group were considered, there was no increase in mortality associated with age, type I neurologic lesion, preoperative hospital stay, or CPB time. However, IAB presented an OR of 5.00 (95% CI 1.33-18.74; *P*=0.0169); dialysis was found to have an OR of 28.15 (95% CI 2.58-307.22; *P*=0.0062), and creatinine variation had an OR of 3.67 (95% CI 1.38-9.78; *P*=0.0091). The details are shown in Table 1.

In the QII group, neither age, CPB time, preoperative hospital stay, IAB, nor dialysis were correlated with any increase in mortality. On the contrary, creatinine variation had an OR of 4.37 (95% CI 1.35-14.06; *P*=0.0133), and the type I neurological lesion had an OR of 73.55 (95% CI 16.20-333.76; *P*<0.0001). More information can be found in Table 2.

When the QIII group was considered, there were no increases in mortality associated with CPB time or age; however, IAB presented an OR of 4.86 (95% CI 1.37-17.21; *P*=0.014), creatinine variation had an OR of 3.18 (95% CI 1.27-7.99; *P*=0.0134), dialysis had an OR of 62.67 (95% CI 5.55-707.74; *P*=0.0008), type I neurological lesion had an OR of 5.85 (95% CI 1.77-19.35; *P*=0.0038), and preoperative hospital stay had an OR of 3.05 (95% CI 1.20-7.72; *P*=0.0182), as detailed in Table 3.

When the QIV group was considered, no increase in mortality was found to be associated with type I neurological lesions or with preoperative hospital stay. On the contrary, IAB presented an OR of 3.88 (95% CI 1.69-8.90; *P*=0.0013), CPB time had an OR of 2.60 (95% CI 1.35-4.97; *P*=0.0039), creatinine variation had an OR of 2.00 (95% CI 1.03-3.86; *P*=0.039), dialysis had an OR of 4.05 (95% CI 1.48-11.06; *P*=0.0062), and age had an OR of 2.27 (95% CI 1.21-4.24; *P*=0.0101). Details can be found in Table 4.

## DISCUSSION

This is an observational study that relied on a database. Despite this design, we cannot underestimate its scientific value, particularly with regard to mortality rates. Most importantly, we identified an increase in mortality rates as high as 112% between the QII group and the AC QIV group. There was no increase in mortality rates among patients aged 65 years or older or whose CPB times were longer than or equal to 115 minutes in the QI, II and III groups. Preoperative hospital stay of 5 days or longer was correlated with increased mortality rates only in the QIII group. Creatinine variation greater than or equal to 0.4 mg/dL increased mortality rates in all groups. Type I neurological lesions increased mortality in the QII group and in the QIII group. Dialysis and IAB did not increase mortality in the QII group.

Increased preoperative creatinine is an independent risk factor known for morbidity and mortality after cardiac surgery^[6-9]^. When AC was considered in isolation in this study, mortality was found to increase as AC increased. Significant differences were found between the QI group and the QIV group, with an OR of 1.72 (95% CI 1.01-3.00; *P*=0.0392). Significant differences were also found between the QII group and the QIV group, with an OR of 2.26 (95% CI 1.31-4.01; *P*=0.0016). These results are detailed in Figure 1. Similar findings were also reported by Miceli et al.^[6]^, who concluded that when creatinine clearance was less than 60 mL/min/1,73 m^2^, mortality rates increased by 12% to 164%.

In our study, IAB insertion increased mortality rates in all AC strata, with the exception of the QII group (*P*=0.22), a difference which was likely due to the low incidence of deaths. The results from Rampersad et al.^[10]^ (meta-analysis with a total of 1,200 patients from 1997 to 2014) go in another direction. They stated that IAB insertion actually decreases mortality rates (RR 0.48, 95% CI 0.30-0.76, *P*<0.01) and major adverse cardiac and cerebrovascular events (RR 0.67; 95% CI 0.54-0.84 *P*<0.001). In a detailed analysis, we can see each paper included in that meta-analysis. Most part of those small papers were inconclusive for mortality and major adverse cardiac and cerebrovascular events (MACCE) and even some with high mortality rates, but when we put them together we can achieve statistic significance. Even if we had enrolled 1,599 patients, an observational study will never have the power to indicate whether a procedure is definitely related to increased mortality. For this, we will need a clinical trial or meta-analysis of clinical trials. Observational papers are very good at making hypothesis and defining the local characteristics of our patients like we did here.

CPB time increased mortality in our study in the QIV group. Otherwise, CPB time between 115 and 188 minutes was not associated with an increase in mortality (188 minutes being the longest CPB time recorded in this study). Taniguchi et al.^[11]^ and Rodrigues et al.^[12]^ agree that longer CPB time is correlated with increased mortality (over 90 and 120 minutes, respectively).

Creatinine variation greater than or equal to 0.4 mg/dL increased the mortality rate in every creatinine stratum. When this variation was associated with QI group, mortality increased by 267% (OR 3.67; 95% CI 1.38-9.78) and, when creatinine variation was associated in the QIV group, mortality increased by 100% (OR 2.00; 95% CI 1.03-3.86). Machado et al.^[8,13]^ and Santos et al.^[5]^ found similar results, reporting increased mortality when creatinine variation was more than 0.3 mg/dL and 0.4 mg/dL, respectively. The consistent increase in mortality when creatinine variation exceeds 0.4 mg/dL is quite important. Until now, this increase was known to be harmful to patients, but the question remained regarding the greater risk between a patient with AC 0.2 mg/dL whose creatinine increased to 0.6 mg/dL and a patient with AC 2.6 mg/dL whose creatinine increased to 3.0 mg/dL. Our results support the conclusion that, when creatinine variation is higher than 0.4 mg/dL, mortality rates increase regardless of the AC value.

In our study, dialysis was associated with increased mortality in almost all AC strata. The only AC stratum in which the difference was not significant was that of QII group, a difference that was likely due to the limited number of patients in this group (*P*=0.06). In the other strata, OR was found between 4.05 and 62.67. These findings are in accordance with those of Chertow et al.^[14]^, who found that acute kidney failure associated with dialysis is an independent risk factor for death, with an OR of 27 (95% CI 22-34).

The prevalence of type I neurological lesion after ONCABG is approximately 2% to 6.1%. It is associated with chronic kidney injury, recent myocardial infarction, cerebrovascular accident, carotid artery disease, peripheral vascular disease, previous cardiac surgery, hypertension, diabetes, atrial fibrillation, preoperative infection, age greater than 75 years, severe or moderate left ventricular dysfunction, CPB time over 120 minutes, and massive transfusion^[15,16]^. According Stamou et al.^[15]^, the incidence of death associated with type I neurological lesions is 4.18 times higher than among those who do not present this type of neurological lesion. Our results determined a mortality rate that was 4.85 to 72.55 times higher, except for the QI group and the QIV group. These groups likely differed because of the small number of patients (*P*=0.20 and *P*=0.63, respectively). Taking into account the severity of this type of lesion, which is generally associated with worse postoperative outcomes, Santos et al.^[5]^ outlines some protective measures to reduce its incidence, including minimal manipulation of the aorta and maintenance of the highest gradient pressure during CPB, especially in patients with a prior history of cerebrovascular accident.

The only group in which preoperative hospital stay was 5 days or longer was the QIII group, with OR 3.05 (95% CI 1.20-7.72), *P*=0.0182. Our findings are in accordance with those of Santos et al.^[5]^. These authors pointed out an increase in mortality with an OR of 1.53 (95% CI 1.03-2.27; *P*=0.03). The hospital stay before surgery may be associated with clinical severity, a correlation which suggests an advanced degree of coronary involvement that may result in increased morbidity and mortality rates in the postoperative period.

Naughton et al.^[17]^ found that age (75 years or older) is an independent risk factor for early mortality (death within fewer than 30 days), with an HR of 2.0 (95% CI 1.28-3.11), but that it is not an independent risk factor for late mortality. In our study, age (65 years or older) was associated with higher mortality rates only in the QIV group. Nevertheless, mortality did not increase in the QI and QII groups, even among patients older than 65 years (the highest age in this study was 88 years old).

No other study in the literature seems to support an association between patients with CPB time longer than 115 minutes or age above 65 years and an AC level less than 1.4 mg/dL. Nevertheless, these results must be evaluated with caution, although not with skepticism. If these results are found to be reproducible by other authors, they may possibly change certain practices in cardiovascular surgery, leading us to rethink the indication of faster ONCABG and the decision to disregard one or more grafts. These results should also lead us to rethink some of the contraindications for this type of surgery, especially for elderly patients. The analyses of these variables in each creatinine stratum by other authors are necessary to confirm or reject our findings.

### Limitations

This study has several limitations. It is based on a large institutional database and results that were collected prospectively, and therefore we are unable to access the influence of any variable outside the database. The observational nature of this study makes it useful for formulating hypotheses, but the results are not strong enough to change routines. Other papers are needed to certify our findings.

## CONCLUSION

Creatinine levels play an important role in ONCABG mortality. The association between selected risk factors and higher AC values leads to a worse prognosis. Conversely, lower AC values were associated with a protective effect, even for elderly patients and with longer CPB times.

**Table:** 

Authors' roles & responsibilities	
MABO	Conception and design of the work; acquisition, analysis, interpretation of data for the work; drafting the work and revising it critically for important intellectual content; final approval of the version to be published
CAS	Interpretation of data for the work; revising it critically for important intellectual content; final approval of the version to be published
ACB	Acquisition, analysis, interpretation of data for the work; final approval of the version to be published
AHD	Acquisition, analysis, interpretation of data for the work; final approval of the version to be published
PHHB	Acquisition, analysis, interpretation of data for the work; final approval of the version to be published
MFG	Conception and design of the work; revising it critically for important intellectual content; final approval of the version to be published
DMB	Conception and design of the work; revising it critically for important intellectual content; final approval of the version to be published
